# Autism, ADHD, and Their Traits in Adults With Bulimia Nervosa and Binge Eating Disorder: A Scoping Review

**DOI:** 10.1002/erv.3177

**Published:** 2025-01-26

**Authors:** Lauren Makin, Elisa Zesch, Adia Meyer, Valeria Mondelli, Kate Tchanturia

**Affiliations:** ^1^ Department of Psychological Medicine Institute of Psychiatry, Psychology & Neuroscience King's College London London UK; ^2^ National Institute for Health and Care Research (NIHR) Maudsley Biomedical Research Centre at South London and Maudsley NHS Foundation Trust King's College London London UK; ^3^ Department of Psychological Medicine Centre for Research in Eating and Weight Disorders (CREW) King's College London London UK; ^4^ Department of Eating Disorders South London and Maudsley NHS Foundation Trust London UK; ^5^ Clinical Psychology Ilia State University Tbilisi Georgia

**Keywords:** binge‐eating, comorbidity, eating disorders, neurodiversity, prevalence

## Abstract

**Objective:**

This review maps existing literature on the prevalence of autism and ADHD in adult patients with Bulimia Nervosa (BN) and Binge Eating Disorder (BED); patient and stakeholder perspectives on this comorbidity; clinical differences in this population; and potential treatment adaptations or adjunct therapies. This is with the aim to inform future research priorities to improve clinical practice.

**Method:**

As pre‐registered, and following PRISMA guidelines, six databases (Embase, MEDLINE via Ovid, PsycINFO, Web of Science, CENTRAL, and Scopus) were searched for studies regarding autism and/or ADHD (diagnosed, probable, or traits) in adult patients with BN or BED. Screening and data extraction were conducted twice independently for each record.

**Results:**

Twenty‐nine studies were included, with 25,416 participants, mostly women (69.3%). Thirteen prevalence studies suggested autism and ADHD are more common in BN or BED than non‐ED populations. One study explored the expert perspectives on autism and ADHD in BED, while 15 studies considered treatment options, mainly medications.

**Conclusion:**

This review highlights a need for more research on the experiences, clinical differences, and non‐medical treatment options for Autistic/ADHD patients with BN or BED. Findings suggest these conditions commonly co‐occur but remain under‐explored in terms of patient‐centred interventions and clinical outcomes.

**Summary**

- Autism and ADHD are likely to be over‐represented in patients with bulimia nervosa (BN) and binge eating disorder (BED).
- No studies currently examine the experiences of these patients and other stakeholders, or the clinical differences between patients with and without autism or ADHD.
- Treatment discussions focus mostly on ADHD medications, without consideration of alternative interventions like psychotherapy and psychoeducation.

## 1 Introduction

Neurodevelopmental conditions, such as autism spectrum disorder (autism) and attention‐deficit/hyperactivity disorder (ADHD), may be more prevalent among individuals with eating disorders (EDs) than in the general population (Nickel et al. 2019; Parsons 2023). This comorbidity presents unique challenges in the clinical presentation, diagnosis, and treatment of EDs in these individuals (Tchanturia 2022). However, research has predominantly focused on autism and anorexia nervosa (AN), with limited attention to ADHD or other EDs, such as bulimia nervosa (BN) and binge eating disorder (BED)(Tchanturia 2022).

Autism is characterised by differences in social interaction, communication, sensory processing, and restrictive, repetitive behaviours (APA 2013), which can impact eating (Kinnaird, Norton, Pimblett, et al. 2019; Nimbley et al. 2022). This paper uses identity‐first language, referring to ‘Autistic individuals’ in line with the preferences of the Autistic community (Bottema‐Beutel et al. 2021; Kenny et al. 2016). Due to wait times and other challenges associated with receiving a formal autism diagnosis, particularly in women (Crane et al. 2018; Lockwood Estrin et al. 2021; NHS 2023), studies have instead often looked at autistic traits or probable autism in patients and found these to still be of clinical utility (Li et al. 2022). When discussing studies, we will make it clear how autism was assessed, but more generally we will use the terms autism and autistic to encompass individuals with both formal autism diagnoses and those presenting with high autistic traits.

Qualitative studies have been key in understanding how autism interacts with EDs (Babb et al. 2021; Brede et al. 2020; Kinnaird, Norton, and Tchanturia 2017; Sedgewick et al. 2022). For example, Autistic individuals with AN reported that feelings of ‘difference’ may have contributed to ED development (Nimbley et al. 2023). Additionally, they felt that their autism‐related needs are often unmet by standard ED treatments (Kinnaird, Norton, Stewart, et al. 2019), leading to increased use of intensive treatment options (Nazar et al. 2018; Nimbley et al. 2024; Tchanturia et al. 2021). Autistic individuals with AN also show differences in clinical presentation, including increased illness severity, earlier onset, and longer duration compared to non‐Autistic individuals (Babb et al. 2022; Zhang et al. 2022). These findings have informed a series of treatment adaptions, called the PEACE pathway (https://www.peacepathway.org/), which reduced admission lengths for patients with AN and autistic traits and saved £22,837 per patient (Tchanturia et al. 2021).

Despite this progress, most research has focused on Autistic patients with AN, potentially overlooking the experiences of Autistic or ADHD patients with BN or BED (Tchanturia 2022). Existing adaptations, like the PEACE pathway, primarily focus on intensive programs, while BN and BED are generally managed in outpatient settings. Given that BN and BED account for almost half of specific ED cases (17% and 23%, respectively) (Hay et al. 2017), it is crucial that research accounts for these conditions.

Although two systematic reviews and one meta‐analysis have investigated autism within ED populations (Huke et al. 2013; Nickel et al. 2019; Sader et al. 2024), none specifically examined BN or BED. Nickel and colleagues (2019) reviewed 17 studies with 5629 participants, finding around 25% of ED patients scored above autism thresholds; however, 13 of these studies focused exclusively on AN, with the remaining four studies including only 12 patients with BN and none with BED. Similarly, Huke and colleagues (2013) reviewed eight studies (four not included by Nickel et al. 2019). None of the four novel studies included BN or BED. Sader and colleagues (2024) focused only on the restrictive ED, ARFID. These reviews demonstrates a lack of clarity regarding available studies and their findings, while highlighting the overall need for further research on autism in BN and BED.

ADHD may also affect ED risk, presentation, and treatment (Cortese, Bernardina, and Mouren 2007; El Archi et al. 2020; Nickel et al. 2019). ADHD is characterised by persistent patterns of inattention, hyperactivity, and impulsivity (APA 2013). Impulsivity may contribute to binge eating or purging behaviours, while inattention could hinder awareness of hunger or satiety (Cortese, Bernardina, and Mouren 2007; Ptacek et al. 2016). Emotional dysregulation may lead to emotional eating as a coping mechanism, and executive function challenges may cause irregular eating patterns (El Archi et al. 2020).

There is currently limited research on the language preferences of ADHD individuals. This paper uses identity‐first language, as outlined for autism, and includes both formally diagnosed individuals and those exhibiting high ADHD traits. We acknowledge that this decision may not be optimal and recognise the need for further research into the preferences of this population regarding how they are referenced, discussed, and studied. As with autism, ED research has often explored ADHD traits and probable ADHD (Kaisari, Dourish, and Higgs 2017), as well as formal ADHD diagnoses (Cortese and Terssari 2017).

In contrast to autism, there is more literature exploring ADHD in relation to BN and BED, with multiple systematic and narrative reviews. Nickel and colleagues (2019) identified seven studies on ADHD prevalence in ED populations, with rates between 1.6% and 18%, though many lacked control groups. This makes it hard to contextualise these findings compared to a general population. In contrast, a meta‐analysis by Nazar and colleagues (2016) only included studies with control groups (*n* = 5 studies), and found elevated ADHD risk in patients with binge eating behaviours, although this was not restricted to patients with full ED syndrome diagnoses. A systematic review by Kaisari and colleagues (2017) found that symptoms of ADHD are associated with disordered eating, including impulsivity being associated with bulimic behaviours. Müller (2014) conducted a review on pharmacological treatments for adults with ADHD and comorbid EDs. These reviews suggest a larger body of literature on ADHD in relation to BN and BED.

However, much of this literature emphasises biological and genetic factors (Seymour et al. 2015) and it is not clear how many studies are useful for clinical practice. For example, a preliminary search revealed few qualitative studies on the perspectives of adults with ADHD and EDs. This suggests that further research may be needed to improve clinical practice for this population.

Sex differences further influence the intersection of autism, ADHD, and EDs, with these conditions often manifesting differently across genders (Breton, Juster, and Booij 2023; Cruz et al. 2024; Martin 2024). Resultantly, autism and ADHD are often underdiagnosed in women and ED research often neglects male patients. This highlights the need to better understand these conditions across genders and ED diagnoses.

In summary, there appears to be a great degree of variation in the amount of research on autism and ADHD in adult patients with BN and BED. To inform future research and practice, a scoping review was deemed appropriate to systematically map existing literature and identify knowledge gaps relevant to the following questions:

1. What is the relative prevalence of autism and ADHD in adults with BN and BED, compared to the general population and other clinical ED populations?
2. What are the experiences and perspectives of Autistic and ADHD adults with BN and BED and other stakeholders?
3. What clinically relevant differences exist between Autistic/ADHD and non‐Autistic/ADHD adults with BN and BED, including in presentation, comorbidities, and treatment outcomes?
4. What treatment adaptions or adjunct therapies exist for Autistic and ADHD adults with BN and BED?

## 2 Methods

A search for any existing scoping or systematic reviews on this topic was conducted on the 6.3.24 in Cochrane Database of Systematic Reviews, PROSPERO, Embase, OVID Medline, APA PsycINFO, and Web of Science. The search strategy used is recorded on the OSF (DOI 10.17605/OSF.IO/S7ATF). Relevant reviews were integrated into this paper's introduction. None were deemed to fulfil the aims of this review.

Aromataris and Munn (2020) guided the study protocol, which was pre‐registered on the OSF (DOI 10.17605/OSF.IO/3JB24), and originally included studies exploring obesity as well as BN and BED. Due to an unexpectedly high yield of results, the protocol was revised to simplify screening, and obesity results will be reported in a separate review. Eligibility criteria were also refined during screening. All changes were reported on the OSF. Tricco et al. (2018) was used as the reporting guideline.

### 2.1 Eligibility Criteria

Studies were included if they: (1) involved human adults (18+) diagnosed with BN or BED, with qualitative studies including carers, family members, healthcare professionals, or experts; (2) assessed or reported diagnosed or probable autism, ADHD, or autistic or ADHD traits. Studies were excluded if they involved only animals, children, other EDs, sub‐clinical ED behaviours, aggregated samples, unidimensional traits of autism/ADHD, solely biological or genetic data, or had insufficient abstract information and non‐English full text. Samples with other comorbidities were not excluded, and no restrictions were placed on sex, ethnicity, location, or publication year. Full eligibility criteria are in Supporting Information S1: Appendix 1.

### 2.2 Information Sources and Search Strategy

LM developed the search strategy in consultation with university librarians and reviewed with all authors. Pilot searches informed the final strategy and eligibility criteria. Terms for EDs (e.g. ‘bulimia’, ‘binge eating disorder’) were combined with neurodevelopmental condition terms (e.g. ‘autism’, ‘ADHD’) using both keywords (e.g., ‘bulimi*’) and database‐specific indexing terms (e.g., MeSH descriptors), while excluding animal studies. Full search strategies for each database are in Supporting Information S1: Appendix 2. Databases used were Embase, MEDLINE via Ovid, PsycINFO, Web of Science, CENTRAL, and Scopus. Reference lists from reviews that passed title‐and‐abstract screening were also searched. Initial searches were conducted on 28.3.24. and final checks on 1.11.24.

### 2.3 Study Selection/Screening

LM, EZ, and AM conducted screening and paper selection. After removing duplicates, titles and abstracts were screened (stage 1). Records passing this stage moved to full‐text screening (stage 2), followed by hand‐searching review articles (stage 3). Discrepancies were discussed within the team and principal author. Interrater reliability was *K* = 0.69, indicating acceptable agreement and crude agreement exceeded our pre‐specified 80% threshold. A follow‐up search was performed by LM to capture new publications.

### 2.4 Data Extraction

Data was extracted using a form developed for this review, available on the OSF (v2).

LM extracted data from all reports, while EZ and AM independently performed a second extraction on all papers. No major discrepancies were noted.

## 3 Results

The initial search yielded 14,895 results, with 7628 duplicates, leaving 7267 articles for screening. Of these, 394 were assessed for eligibility, and 27 met the inclusion criteria; 68 reports were removed to be screened separately for an obesity‐focused review. Main exclusion reasons included aggregated data across ED diagnoses, absence of autism/ADHD assessment, and biological‐only data. A follow‐up search produced 1296 results were yielded in the follow up search, of which 677 were duplicates; 13 articles were assessed for eligibility and two met inclusion criteria, with 10 removed for the obesity review. A PRISMA flow chart is in Supporting Information S1: Appendix 3 (Haddaway et al. 2020).

### 3.1 Overview of Studies

Studies spanned 1997 to 2024 (Supporting Information S1: Appendix 4). One study included data from 12 countries (R. C. Kessler et al. 2013). Excluding this, studies were mainly from North America (*n* = 12), particularly the USA (*N* = 11), and Western Europe (*n* = 12). Two studies were from Asia (India and Japan), one was from South America (Brazil), and one was from Australia.

Of the 29 studies included, 13 examined prevalence of autism, ADHD, or their traits in BN and BED (*n* = 25,328). Five of these were general population cohort studies (four of which used overlapping datasets; *n* = 22,787) and eight used clinical populations (seven studies on ED patients, one study on obesity patients; *n* = 2541). One study by Bray and colleagues (2022) considered the qualitative perspectives of BED experts regarding the relevance of autism and ADHD to BED (*n* = 14). No studies compared clinical differences in patients with BN or BED with versus without autism or ADHD. Fifteen studies explored interventions for Autistic and ADHD patients with BN and BED (*n* = 74). Thirteen of these are case reports (*n* = 23), one was an open‐label trial (*n* = 41), and one was an experimental study with a between‐ and within‐subjects design (*n* = 10). Table 1 maps these papers.

**TABLE 1:** Mapping the number of papers investigating autism or ADHD in BN or BED across the four topics of this review: prevalence, experiences and perspectives, clinical differences, and interventions.

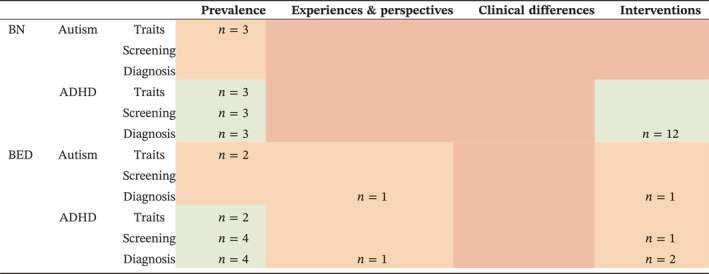

Abbreviations: ADHD, attention‐deficit/hyperactivity disorder; BN, bulimia nervosa; BED, binge eating disorder; *n*, number of studies.

### 3.2 Characteristics of Study Participants

These studies included 25,416 adult participants, mostly women (69.3%). Eight studies reported ethnicity of participants (*n* = 4826; white = 55.4%). Further participant characteristics are described in Tables 2, 3, 4 (cohort prevalence studies), 3 (clinical sample prevalence studies), and 4 (case reports and intervention studies), and Supporting Information S1: Appendix 5 (qualitative study). Notably, ADHD medication status was not specified in any prevalence studies despite its known effects on eating behaviours.

**TABLE 2:** Descriptions of cohort studies reporting prevalence of ADHD in individuals with BN and BED.

	Study	Country	Sample (age, years)	n	ADHD measure			n/%	Gender *Women*	Ethnicity *White*	ADHD	Statistics	
						ED						Ratio (95% CI)	*p* value
1.	Appolinario et al. (2022)	Brazil	Household survey (18–60)	2297	ASRS‐6							*Current ADHD*	
						Current BN		*n* = 17 0.7%	*n* = 15 88.2%	*n* = 6 35.3%	*n* = 8 54.3%	RR^a^ 13.1 (6.1–28.1)	—
												RR^b^ 13.8 (6.8–28.0)	
						Current BED		*n* = 29 1.4%	*n* = 24 82.8%	*n* = 6 20.7%	*n* = 12 35.8%	RR^a^ 8.8 (4.1–18.7)	—
												RR^b^ 10.3 (4.3–24.7)	
2.	Brewerton and Duncan (2016)	USA	NCS‐R^c^ (18–44)	1686	DSM‐IV CIDI (lifetime)							*Lifetime ADHD*	
						BN	Lifetime	*n* = 36	*n* = 34 94.4%	—	*n* = 10 33.17%	*Women*: OR^d^ 7.93 (2.75–22.85)	<0.001
											*n* = 1 66.94%	*Men*: OR^d^ 18.18 (1.39–238.40)	0.006
							12‐month	*n* = 11	*n* = 11,100%	—	*n* = 6 56.69%	*Women*: OR^d^ 21.15 (3.76–118.98)	<0.001
						BED	Lifetime	*n* = 65	*n* = 46 70.8%	—	*n* = 8 17.08%	*Women*: OR^d^ 3.01 (1.14–7.95)	0.015
											*n* = 6 25.35%	*Men*: OR^d^ 2.93 (0.98–8.76)	0.033
							12‐month	*n* = 702	*n* = 694 98.9%	—	*n* = 4 19.29%	*Women*: OR^d^ 3.57 (1.06–12.09)	0.034
											*n* = 4 45.90%	*Men*: OR^d^ 6.47 (0.98–8.76)	0.001
3.	Hudson et al. (2007)	USA	NCS‐R^c^ (18–44)	1672	DSM‐IV CIDI							*Lifetime ADHD*	
						Lifetime BN		1.0%	—	—	34.9%	OR^e^ 8.4 (3.2–22.5)	≤0.05
						Lifetime BED		2.8%	—	—	19.8%	OR^e^ 3.0 (1.4–6.3)	≤0.05
4.	R. C. Kessler et al. (2013)	12 countries^f^	WMH inc. NCS‐R^c^ (18+)	12,413	DSM‐IV CIDI							*Lifetime ADHD*	
						Lifetime BN		1.0%	—	—	14.8%	OR^g^ 5.8 (2.9–11.5)	<0.05
						Lifetime BED		1.9%	—	—	10.2%	OR^g^ 3.9 (1.8–8.7)	<0.05
5.	Ziobrowski, Brewerton, and Duncan (2018)	USA	NCS‐R^c^, NSAL (18–44)	4719	DSM‐IV CIDI				52.1%	55.5%			
						BN	Lifetime	1.2%			—	*Lifetime ADHD*	
												OR^a^ 5.78 (2.59–12.90	—
												OR^e^ 7.30 (3.40–15.67)	
												OR^h^ 2.52 (1.04–6.10)	
							12‐month	0.4%			—	*12‐month ADHD*	
												OR^a^ 21.41 (5.47–83.71)	—
												OR^e^ 28.24 (6.33–126.01)	
												OR^h^ 5.04 (1.15–22.09)	
						BED	Lifetime	2.5%			—	*Lifetime ADHD*	
												OR^a^ 2.60 (1.33–5.09)	—
												OR^e^ 2.55 (1.25–5.24)	
												OR^h^ 1.42 (0.74–2.74)	
							12‐month	1.0%			—	*12‐month ADHD*	
												OR^a^ 4.46 (1.81–10.95)	—
												OR^e^ 4.53 (1.82–11.24)	
												OR^h^ 1.65 (0.67–4.04)	

Abbreviations: ADHD, attention‐deficit/hyperactivity disorder; ASRS‐6, six‐item Adult ADHD Self‐report Scale; BED, binge eating disorder; BN, bulimia nervosa; CI, confidence interval; DSM‐IV CIDI, Composite International Diagnostic Interview for the fourth edition of the Diagnostic and Statistical Manual of Mental Disorders; *n*, number of participants; NCS‐R, replication of the National Comorbidity Survey; NSAL, National Survey of American Life; OR, odd's ratio; RR, relative risk; USA, United States of America; WMH, World Mental Health Survey.

^a^ Unadjusted model.

^b^ Adjusted for gender, race, & BMI.

^c^ Samples overlap as NCS‐R was used by three studies.

^d^ Adjusted for age & race.

^e^ Adjusted for age, race, & sex.

^f^ Belgium, Brazil, France, Germany, Italy, Mexico, Netherlands, Northern Ireland, Portugal, Romania, Spain, United States of America.

^g^ Adjusted for age, sex, & country.

^h^ Adjusted for age, race, sex, and lifetime psychiatric disorders.

**TABLE 3:** Descriptions of studies reporting prevalence of autism and ADHD in clinical samples.

	Study	Country	Design	Sample	Age, years *Mean ± SD (range)*	Gender *Women (%)*	Ethnicity *White (%)*	Neurodiversity *(measure)*	ED	Findings *Score mean ± SD/prevalence n*	*p‐*value
6.	Dell’Osso et al. (2018)	Italy	Case‐control	298 (138 ED, 160 controls)	32.1 ± 10.1	76.2	—	Autistic traits *(AdAS Spectrum)*. *Excluded those with diagnosed autism (SCID‐5)*	34 BN	52.8 ± 24.7	Vs No ED, < 0.05
					40.5 ± 16.7				34 BED	52.1 ± 20.5	Vs No ED, < 0.05
					29.3 ± 11.0				46 AN‐R	62.3 ± 26.7	*F* = 21.77, *p* < 0.001 for comparing all 5 groups
					27.8 ± 6.8				24 AN‐BP	52.5 ± 17.4	
					26.5 ± 9.1				160 No ED	33.2 ± 20.1	
7.	Ferre et al. (2017)	Spain	Cross‐sectional	89 ED	*ADHD*: 32.8 ± 9.9; *Non‐ADHD*: 30.8 ± 9.8	95.5	—	ADHD screening *(ASRS‐6)*	31 BN	*n* = 18 (58.1%)	TS (4) = 5.066, *p* = 0.28
									12 BED	*n* = 8 (66.7%)	
									9 AN‐R	*n* = 2 (22.2%)	
									13 AN‐BP	*n* = 7 (53.8%)	
									24 Unspecified ED	*n* = 11 (45.8%)	
8.	Gesi et al. (2017) ^a^	Italy	Pilot study	346 (241 ED, 105 controls)	—	—	—	Autistic traits *(AQ‐50*, *AdAS Spectrum*, *RAADS‐14)*	41 BN	—	Vs No ED < 0.001
									42 BED	—	Vs No ED < 0.001
9.	Iwasaki et al. (2013)	Japan	Case‐control	143 (84 ED, 59 controls)	*ED*: 24.0 ± 6.6; *Controls*: 20.0 ± 1.4	100	—	Autistic traits *(AQ‐50*, *Japanese version)*	13 BN‐P	21.92 ± 8.08	N.S versus No ED;
									10 BN‐NP	24.10 ± 6.79	N.S Versus No ED
									35 AN	26.06 ± 5.98	Vs overall BN < 0.001
									59 No ED	22.92 ± 5.67	—
10.	Muller et al. (2012)	Germany	Cross‐sectional	90 obesity	37.9 ± 11.2 (18–66)	72.2	—	ADHD screening *(WURS‐k*, *ADHD‐SR)*	22 BED	3 (13.6%)	N.S
									68 No BED	5 (7.4%)	
11.	Sala et al. (2018)	France	Cross‐sectional	73 ED	28.07 ± 7.3 (17–50)	100	—	ADHD dx and traits *(DSM‐IV‐TR*; *WURS*; *BADDS)*	8 BN	*n* = 1 (12.5%); WURS 36.88 ± 25.08, BADDS 53.00 ± 23.02	*F* (2.70) = 8.150, *p* < 0.001, comparing WURS total for all three groups
									37 AN‐R	*n* = 3 (8.1%); WURS 21.68 ± 16.31, BADDS 42.92 ± 24.29	
									28 AN‐BP	*n* = 9 (32.1%); WURS 39.21 ± 18.12, BADDS 58.96 ± 23.29	
12.	Svedlund et al. (2017)	Sweden	Cross‐sectional	1094 ED	27.7 ± 8.7 (18–70)	100	—	ADHD screening and traits *(ASRS‐6)*	421 BN	*n* = 156 (37.1%); ASRS median 12	Vs AN‐R: *U* = 8942, *p* = 0.000, *r* = 0.23
									91 BED	*n* = 25 (27.5%); ASRS median 10	Vs AN‐R: *U* = 2187, *p* = 0.002, *r* = 0.25
									AN‐R	*n* = 12 (17.6%); ASRS median 7	χ2 (5, *n* = 1094) = 15.30, *p* = 0.009, Cramer's V = 0.12 across all 6 groups
									AN‐BP	*n* = 13 (35.1%); ASRS median 11	
									EDNOS‐R	*n* = 38 (25.7%); ASRS median ‐	
									EDNOS‐BP	*n* = 102 (31.0%); ASRS median 10	
13.	Svedlund et al. (2021)	Sweden	Prospective	408 ED	28.3 ± 9.3 (18–70)	100	—	ADHD traits *(ASRS‐6)*	108 BN (recovered at 1 year)	ASRS median baseline 11; follow up 9	Baseline versus follow up *Z* = −3.9, *p*<=0.000
									42 BN (not recovered)	Baseline 11; follow up 12.5	Baseline versus follow up *Z* = −0.7, *p* = n.s
									32 BED (recovered)	Baseline 10; follow up 10	Baseline versus follow up *Z* = −1.3, *p*<=n.s
									11 BED (not recovered)	Baseline 12; follow up 10	Baseline versus follow up *Z* = −0.2, *p* = n.s

Abbreviations: AdaS Spectrum, Adult Autism Subthreshold Spectrum; ADHD, attention‐deficit/hyperactivity disorder; ADHD‐SR, The German ADHD Rating Scale; AN‐BP, anorexia nervosa, binge‐purge subtype; AN‐R, anorexia nervosa, restrictive subtype; AQ‐50, The 50‐item Autism Spectrum Quotient; ASRS‐6, 6‐item Adult ADHD Self‐report Scale; BADDS, The Brown Attention Deficit Disorder Scale; BED, binge eating disorder; BN, bulimia nervosa; DSM‐IV CIDI, Composite International Diagnostic Interview for the fourth edition of the Diagnostic and Statistical Manual of Mental Disorders; DSM‐IV‐TR, The text revision of the fourth edition of the Diagnostic and Statistical Manual of Mental Disorders; ED, eating disorder; EDNOS‐BP, eating disorder not otherwise specified, binge‐purge subtype; EDNOS‐R, eating disorder not otherwise specified, restrictive subtype; *n,* number of participants; RAADS‐14, 14‐item Ritvo autism and Asperger diagnostic scale; SCID‐5, he Structured Clinical Interview for the fifth edition of the Diagnostic and Statistical Manuel of Mental Disorders; SD, standard deviation; vs, versus; WURS, 61‐item Wender Utah Rating Scale; WURS‐k, 25‐item Wender Utah Rating Scale.

^a^ Abstract‐only.

**TABLE 4:** Summary of prevalence findings.

	BN	BED
Autism	Autistic traits were significantly elevated in patients with BN compared to healthy controls in two out of three studies. Autistic traits were more elevated in patients with AN(/‐R) than in those with BN.	Autistic traits were significantly elevated in patients with BED compared to healthy controls.
ADHD	Risk of lifetime and 12‐month ADHD was significantly raised in individuals with lifetime and 12‐month BN compared to those without BN, even when adjusting for demographics and other psychiatric disorders. 15% of individuals with lifetime BN had ADHD. ADHD positive screening rates did not significantly differ across patients with BN (37%–58%) and other EDs (18%–54%). Childhood and current ADHD traits were higher in BN than in AN‐R. Patients with BN who had recovered at 1 year follow up had significantly lower ADHD traits than they did at baseline.	Risk of lifetime and 12‐month ADHD was significantly raised in women with lifetime and 12‐month BED compared to those without BED even when adjusting for demographics, but not in men or when adjusting for other psychiatric disorders. 10% of individuals with lifetime BED had ADHD. ADHD positive screening rates did not significantly differ across patients with BED (14%–68%) and other EDs (18%–54%), or control participants in obese populations (7%). Current ADHD traits were higher in BED than in AN‐R. ADHD scores did not significantly change from baseline to 1 year follow up in patients with BED, regardless of whether they had recovered or not.

Abbreviations: ADHD, attention‐deficit/hyperactivity disorder; AN, anorexia nervosa; AN‐R, anorexia nervosa, restrictive subtype; BN, bulimia nervosa; BED, binge eating disorder; ED, eating disorders.

### 3.3 Measures

#### 3.3.1 Autism

Three prevalence studies focused on autistic traits, not diagnosis (Dell'Osso et al. 2018; Gesi et al. 2017; Iwasaki et al. 2013). Dell’Osso and colleagues (2018) excluded full diagnoses using the Structured Clinical Interview for DSM‐5 Disorders (SCID‐5) (First et al. 2016), and used the Adult Autism Subthreshold Spectrum (AdAS Spectrum) (Dell'Osso et al. 2017) for traits. Iwasaki and colleagues (2013) used the Japanese version of the full autism‐spectrum quotient (AQ‐50) (Baron‐Cohen et al. 2001; Wakabayashi et al. 2004), and Gesi and colleagues (2017) used a combination of both as well as the 14‐item screener of the Ritvo autism and Asperger diagnostic scale (RAADS‐14 Screen) (Eriksson, Andersen, and Bejerot 2013). The only autism case report used SCID‐5, AQ‐50, AdAS Spectrum, and the full 80‐item version of the Ritvo Autism and Asperger Diagnostic Scale (RAADS‐R) (Ritvo et al. 2008) in Italian to make a diagnosis (Carmassi et al. 2019).

#### 3.3.2 ADHD

Most general population studies used semi‐structured interviews for ADHD diagnosis (Brewerton and Duncan 2016; Hudson et al. 2007; R. C. Kessler et al. 2013; Ziobrowski, Brewerton, and Duncan 2018), specifically the DSM‐IV Composite International Diagnostic Interview (DSM‐IV CIDI) (R. Kessler and Ustun 2004). However, Appolinario and colleagues (2022) instead used a self‐administered questionnaire, the six‐item version of the Adult ADHD Self‐Report Scale (ASRS‐6)(R. C. Kessler et al. 2005), which is a screening scale, as a proxy for clinical diagnosis.

Only one prevalence study on a clinical population, by Sala and colleagues (2018), used clinical interviews for ADHD diagnosis, based on the Diagnostic and Statistical Manual of Mental Disorders, 4th edition‐Text Revision (DSM‐IV‐TR) criteria (APA 2000). This diagnosis was integrated by a retrospective instrument, the 61‐item Wender Utah Rating Scale (WURS) (Ward 1993) to help assess adult's descriptions of their childhood behaviour. The Brown Attention Deficit Disorder Scale (BADDS) (Brown 1996) was also administered to assess persisting ADHD symptoms in adulthood. Results from these were also reported as continuous trait scores.

The other four prevalence studies on clinical populations used screening measures. Three used the measure dichotomously and reported positive screening as probable diagnosis (Ferre et al. 2017; Müller et al. 2012; Svedlund et al. 2017), whereas the fourth used the measure as a continuous scale (Svedlund et al. 2021). Three of these studies used the ASRS‐6; two used this scale dichotomously (Ferre et al. 2017; Svedlund et al. 2017), one used it continuously (Svedlund et al. 2021). Whereas Muller and colleagues (2012) used alternative self‐administered questionnaires (dichotomously): the German ADHD Rating Scale (ADHD‐RS) (Rösler 2008) to screen for Adult ADHD and the German version of short 25‐item of the WURS (WURS‐k) (Rösler 2008) to retrospectively screen for childhood ADHD. Only participants who screened positively on both scales were counted as probable ADHD cases.

Thirteen of the case reports and intervention studies used ADHD clinical diagnoses, made mostly using the DSM‐4 or DSM‐IV‐TR. Ioannidis and colleagues (2014) used the Diagnostic Interview for ADHD in Adults (DIVA 2.0) and Keshen and Ivanova (2013) used the Weiss Symptom Record (WRS) to support their clinical diagnosis. Sokol et al. (1999) was the only study to consider ADHD symptoms instead of diagnosis, using a modified Conners Adult ADHD Rating Scale (CAARS) (C. K. Conners 1969). Ruiz and colleagues (2019) used the Conners' Continuous Performance Test II (CPT‐II) (K. C. Conners 2000) to assess ADHD symptoms before and after treatment. Griffiths and colleagues (2024) used the ASRS‐18 to screen for ADHD as well as reporting pre‐existing diagnosed cases.

### 3.4 Findings From Papers

#### 3.4.1 Prevalence

Table 2 summarises cohort prevalence studies on ADHD in BN and BED. Appolinario and colleagues (2022) found that in the general population of Rio de Janeiro in Brazil, relative risk of current probable ADHD was increased in both individuals with current BN, and current BED, compared to those without current BN or BED, even when adjusting for gender, race, and BMI, with 54% of individuals with BN and 36% of individuals with BED screening positive for ADHD. Four studies with overlapping USA samples found that risk of 12‐month and lifetime ADHD was significantly raised in individuals with 12‐month and lifetime BN or BED compared to those without BN or BED (Ziobrowski, Brewerton, and Duncan 2018), even when adjusting for demographics (Hudson et al. 2007; R. C. Kessler et al. 2013; Ziobrowski, Brewerton, and Duncan 2018). When stratified by gender, Brewerton and Duncan (2016) found risk was still elevated in both women or men with lifetime BN, women with 12‐month BN, women with lifetime BED or 12‐month BED, but not men with lifetime BED (*n* = 19) or 12‐month BED (*n* = 8). No men in the sample had 12‐month BN. However, when other psychiatric disorders were controlled for, Ziobrowski and colleagues (2018) found 12‐month or lifetime ADHD risk was no longer elevated in individuals with 12‐month or lifetime BED, although the association remained in individuals with 12‐month and lifetime BN. In their sample of 12 countries, Kessler and colleagues (2013) found 15% of individuals with lifetime BN, and 10% of individuals with lifetime BED had ADHD.

Table 3 summarises studies reporting prevalence of autism and ADHD in clinical samples. Three studies looked at autistic traits in clinical ED samples. Two found autistic traits were significantly elevated in Italian patients with BN and BED compared to healthy controls (Dell'Osso et al. 2018); *p* < 0.05) (Gesi et al. 2017); *p* < 0.001). Dell’Osso and colleagues (2018) also looked at Italian patients with AN‐R and AN‐BP and found there to be a significant difference overall in trait scores across BN, BED, AN‐R, AN‐BP, and the control group (*p* < 0.001). Autistic scores for patients AN‐R were highest (mean 62), and scores for the control group were lowest (mean 33), with BN (mean 53) and BED (mean 52) in the middle. Iwasaki and colleagues (2013) found no significant differences between autistic trait scores in two sub‐groups of female Japanese patients with BN‐P (mean 22) and BN‐NP (mean 24) compared to healthy controls (mean 23). This study also found autistic traits to be significantly higher in female Japanese patients with AN (mean 26) than in those with BN (two BN sub‐groups collapsed; *p* < 0.001).

Three studies compared ADHD positive screening rates in clinical populations (Ferre et al. 2017; Müller et al. 2012; Svedlund et al. 2017). Two studies found that ADHD rates did not significantly differ across different ED diagnoses, with 37%–58% of patients with BN, 28%–67% of those with BED, and 18%–22% of those with AN‐R screening positive for ADHD (Ferre et al. 2017; Svedlund et al. 2017). Muller and colleagues (2012) found that within a German clinically obese population, ADHD positive screening rates were not significantly elevated in those with BED (14%) compared to those without BED (7%).

Three studies looked at ADHD traits in female clinical populations (Sala et al. 2018; Svedlund et al. 2017, 2021). Sala and colleagues (2018) compared childhood ADHD traits across female French patients with BN (mean 37), AN‐R (mean 22), AN‐BP (mean 39) and found a significant difference between groups (*p* < 0.001). AN‐R had the lowest score. Similarly, Svedlund and colleagues (2017) found current ADHD traits were significantly elevated in both female Swedish patients with BN (median 12) and those with BED (median 10) compared to those with AN‐R (median 7; *p* < 0.01). Additionally, Svedlund and colleagues (2021) explored changes in ADHD traits in female Swedish patients with BN or BED. They compared changes in ADHD trait scores from baseline to follow up between patients who had recovered from their ED against patients who had not recovered across the same timepoint. ADHD traits were significantly lower at follow up (median 9) compared to baseline (median 11) in patients with BN who had recovered at follow up (*p* < = 0.000). Changes in ADHD rates at follow up were non‐significant in patients with BN who had not recovered at follow up and were non‐significant in both recovered and non‐recovered BED groups. Table 4 summarises and maps the findings of all included prevalence studies.

#### 3.4.2 Experiences and Perspectives

Bray and colleagues (2022) evaluated the opinions of 14 BED experts, including seven researchers and six clinicians and healthcare administrators (information was not available for one participant). No expert thought autism was relevant to BED. Two experts mentioned autism in relation to ADHD, BED, and sensory input. Thirteen experts considered ADHD to be relevant to BED. Five discussed the extent of this relevance. Six experts described frequent comorbidity between the ADHD and BED. Seven experts discussed the potential nature of this relationship, including relevant mechanisms. Seven experts discussed the use of stimulant medication with mixed views. Four participants discussed issues around detecting and treating this comorbidity. Three participants reported seeing a lot of untreated ADHD in patients with BED. Three participants reported benefits to patients if they can get diagnosed with ADHD when applicable. Full study characteristics are summarised in Supporting Information S1: Appendix 5.

#### 3.4.3 Clinical Differences

No studies looked at clinical differences between Autistic or ADHD patients with BN and BED and those without autism or ADHD.

#### 3.4.4 Interventions

Table 5 summarises case reports and intervention studies. A map of findings from these studies is in Supporting Information S1: Appendix 6. Carmassi and colleagues (2019) detail a case report of a 35‐year‐old female patient with BED, obesity, and other comorbidities who was receiving inpatient pharmacological treatment for depressive symptoms. After a diagnosis of autism was made, she was switched from amitriptyline with sertraline (50 mg) and perphenazine with aripiprazole (5 mg), showing a significant clinical global improvement.

**TABLE 5:** Descriptions of case report and intervention studies.

	Study	Country	Design	Sample (ethnicity, gender)	Age	Comorbidities	ED	Neurodiversity diagnosis *(measure/diagnosis)*	Intervention	Findings
15.	Bhat et al. (2023)	India	Case	1 female	25	Trichotillomania, major depressive disorder	BN	ADHD *(clinical team)*	Methylphenidate, lisdexamfetamine, Escitalopram 10 mg, CBT for habit reversal	Symptom reduction and coping with impulsive behaviours; Partial improvement with ongoing follow‐up for adjustments in treatment.
16.	Carmassi et al. (2019)	Italy	Case	1 female	35	Severe obesity, bipolar disorder, other comorbidities	BED	Autism *(AQ‐50*, *RAADS‐R*, *AdAS Spectrum)*	Switched from amitriptyline with sertraline 50 mg and perphenazine with aripiprazole 5 mg	Significant clinical global improvement
17.	Drimmer (2003)	USA	Cases	2 females	31	—	BN	ADHD (*DSM‐4)*	Adderall 10 mg	ADHD and BN improved significantly.
					42	Depression	BN	ADHD (*DSM‐4)*	Methylphenidate 20 mg;	Bingeing ceased and food cravings diminished
18.	Dukarm (2005)	USA	Cases	4 white females	19	Graves' disease, thyroid ablation	BN	ADHD *(DSM‐IV‐TR)*	Dextroamphetamine sulphate 15 mg	Complete abstinence from binge eating and purging
					18	—	BN	ADHD *(DSM‐IV‐TR)*	Dextroamphetamine sulphate 10 mg	
					21					
					24	Hypothyroidism, depression	BN	ADHD *(DSM‐IV‐TR)*		
19.	Farber (2009)	USA	Case	1 female	20	Generalised anxiety disorder, panic attacks, underactive thyroid	BN	ADHD *(confirmed by neuropsychologist)*	Audio‐visual entrainment (already on Ritalin)	Entrainment improved ADHD symptoms enough Ritalin dose was decreased
20.	Griffiths et al. (2024)	Australia	Open‐label trial	40 females; 1 male (53.7% white)	26.6 ± 5.5 (18–40)		BED	ADHD (*n* = 5) and probable ADHD (*n* = 18; *ASRS‐18)*	Lisdexamfetamine 30 mg	No change in overall ADHD symptoms in those with probable ADHD (*n* = 14), though decrease in inattentive symptoms. 75% of patients with diagnoses ADHD (*n* = 4) achieved clinically relevant treatment response (>70% reduction in binge days per week).
21.	Guerdjikova and McElroy (2013)	USA	Case	1 white female	32	Bipolar I disorder, substance dependences, panic disorder	BN	ADHD *(clinical diagnosis)*	Methylphenidate 30 mg	Complete remission of BN
22.	Ioannidis, Serfontein, and Müller (2014)	UK	Case	1 female	23	—	BN	ADHD (*ASRS‐18*, *DIVA 2*.*0*.)	Dextroamphetamine methylphenidate XR 18 mg	Reduced binge/purges, extinguished ADHD symptoms; EDE‐Q score from 5.6 out of 6 at admission to 2.14 at discharge.
23.	Jacquemet et al. (2021) ^a^	France	Cases	1 female	50	—	BED	ADHD *(clinical diagnosis)*	Methylphenidate	Reduction on binge episodes, decreased in severity of pathology, weight loss
				1 male	30					
24.	Jeremias et al. (2024)	Portugal	Case	1 female	32	—	BN	ADHD *(clinical diagnosis)*	Fluoxetine 60 mg and topiramate 50 mg, lisdexamfetamine 30 mg	No improvement from fluoxetine and topiramate; lisdexamfetamine led to improvement (reduction in ADHD symptoms and in binging and purging episodes) within 2 days
25.	Keshen and Ivanova (2013)	Canada	Cases	5 females	34	—	BN	ADHD *(clinical interview*, *WSR)*	Adderall XR 40 mg	Full remission of BN, some side effects resolved with psychosocial adjustments
					20	—	BN	ADHD *(clinical interview*, *WSR)*	Adderall XR 40 mg	Improvement in concentration, substantial decrease in binging and purging
					23	Alcohol abuse disorder, borderline personality traits	BN	ADHD *(clinical interview*, *WSR)*	Adderall XR 40 mg	Significant improvement in ADHD symptoms and a virtual full remission of binging and purging, significant weight loss. Terminated treatment and relapsed.
					22	Borderline personality traits	BN	ADHD *(clinical interview*, *WSR)*		Remission of BN, some adverse effects resolved with quetiapine and psychosocial adjustments
					32	—	BN	ADHD *(clinical interview*, *WSR)*	Dexedrine spansules 20 mg, then generic methylphenidate	Improvements in concentration and anxiety, decreased urge to purge
26.	Ruiz et al. (2019) ^a^	Spain	Case	1 Hispanic female	18	—	BN	ADHD and symptoms *(CPT‐II)*	Lisdexamfetamine	Reduction in bingeing episodes, attentional and emotional improvement (Conners' CPT‐II)
27.	Santarpia (2008) ^b^	USA	Experiment	10 females (5 intervention group, 5 control)	18+	—	BN	ADHD *(clinical diagnosis)*	20 sessions of neurofeedback + traditional treatment for BN	No brain wave changes, statistically significant reductions in 2 of 6 ED symptoms (EDI‐3), other positive outcomes reported
28.	Schweickert, Strober, and Moskowitz (1997)	USA	Case	1 white female	25	—	BN	ADHD *(by paediatrician)*	Methylphenidate 5 mg	ADHD symptoms improved, cessation of bingeing
29.	Sokol et al. (1999)	USA	Case	2 females	20	Cluster B traits	BN	ADHD symptoms *(modified CAARS)*	Methylphenidate 10 mg	Decrease in ADHD and ED symptoms (conners; EAT)
					38	Cluster B traits	BN	ADHD symptoms *(modified CAARS)*	Methylphenidate 20 mg	

Abbreviations: AdaS Spectrum, adult autism subthreshold spectrum; ADHD, attention‐deficit/hyperactivity disorder; AQ‐50, The 50‐item Autism Spectrum Quotient; ASRS‐18, 18‐item Adult ADHD Self‐report Scale; BN, bulimia nervosa; BED, binge eating disorder; ED, eating disorder; CAARS, conners adult ADHD rating scale; CBT, cognitive behavioural therapy; CPT‐II, Conners' continuous performance test II; DIVA 2.0, diagnostic interview for ADHD in adults; DSM‐IV, the fourth edition of the diagnostic and statistical manual of mental disorders; DSM‐IV‐TR, The text revision of the fourth edition of the diagnostic and statistical manual of mental disorders; EAT, eating attitudes test; EDI‐3, eating disorder inventory‐3; *n,* number of participants; RAADS‐R, 80‐item ritvo autism and asperger diagnostic scale; WRS, the weiss symptom record ADHD rating scale; USA, United States of America.

^a^ Abstract‐only.

^b^ Dissertation.

Ten case reports detailed 19 female patients with BN and ADHD (Bhat et al. 2023; Drimmer 2003; Dukarm 2005; Farber 2009; Guerdjikova and McElroy 2013; Ioannidis, Serfontein, and Müller 2014; Jeremias et al. 2024; Keshen and Ivanova 2013; Ruiz et al. 2019; Schweickert, Strober, and Moskowitz 1997; Sokol et al. 1999). Eighteen of these patients were treated with ADHD stimulant medications, most commonly methylphenidate (*n* = 7) or Adderall (*n* = 5). These were largely reported as effective in reducing or extinguishing either BN symptoms, ADHD symptoms, or both. One patient was also treated with audio‐visual entrainment in order to successfully reduce their Ritalin dose (Farber 2009). The only experimental study used neurofeedback on female patients with BN (Santarpia 2008). This study reported positive outcomes, including statistically significant reductions in two out of six ED symptoms, measured by the EDI‐3, but no changes in brain wave measures.

Jacquemet and colleagues (2021) reported two patient cases (one male) with ADHD and BED who were both treated with methylphenidate. This led to weight loss, decreased severity of pathology, and reduction in binge eating episodes. Griffiths and colleagues (2024) ran a secondary analysis on an open trial of lizdexamfetamine in 41 patients with BED. Three of the five patients with ADHD diagnoses achieved clinically relevant treatment responses (>70% reduction in binge days per week). Across the 14 patients analysed who screened positively for ADHD, there was no change in overall ADHD symptoms, although there was a reduction in inattentive symptoms (*p* = 0.018).

## 4 Discussion

This scoping review assessed the literature on autism and ADHD in adults with BN and BED to identify gaps relevant to clinical practice. The findings indicate a lack of research on this topic, especially regarding autism in these populations.

### 4.1 Autism in BN and BED

Few studies investigated the prevalence of autism in patients with BN and BED, and results were mixed. Two studies found significantly elevated autistic traits in patients with BN and BED compared to non‐ED populations (Dell'Osso et al. 2018; Gesi et al. 2017). However, one study did not find significant difference in autistic traits between BN patients and controls (Iwasaki et al. 2013). Additionally, two of these studies also found that autistic traits were significantly lower in BN and BED than in AN, particularly the restrictive sub‐type (Dell'Osso et al. 2018; Iwasaki et al. 2013). This might suggest that autism is overrepresented in BN and BED, though to a lesser degree than in AN. Notably, BED researchers and healthcare professionals did not think autism was relevant to BED (Bray et al. 2022). No studies addressed clinical differences between Autistic and non‐Autistic patients with BN or BED. One case report reported positive outcomes for an Autistic patient with BED treated for depression with sertraline and aripiprazole (Carmassi et al. 2019). Overall, there remains a substantive gap in understanding autism in BN and BED.

Even if autism is not markedly overrepresented in BN and BED, Autistic patients within these groups may still face unique challenges. Research on autism in AN suggests that autism can affect ED symptoms, presentation, and treatment outcomes (Babb et al. 2022; Nimbley et al. 2023). It is likely that Autistic individuals with BN and BED also experience distinct challenges linked to sensory, cognitive, and social processing differences (Kinnaird, Norton, Pimblett, et al. 2019; Nimbley et al. 2022). Yet, this review found little exploration of these differences, and no adjunct therapies were proposed. The absence of autism comorbidity awareness among BED healthcare professionals (Bray et al. 2022) also suggests a gap in current training.

### 4.2 Prevalence of ADHD in BN and BED

Compared to autism, these is more literature on ADHD in BN and BED. General population studies are limited (and mostly use overlapping USA datasets), but findings consistently showed a heightened ADHD risk in individuals with BN and BED (Brewerton and Duncan 2016; Hudson et al. 2007; R. C. Kessler et al. 2013; Ziobrowski, Brewerton, and Duncan 2018). While Brewerton and Duncan (2016) found this risk reduced in males, small sample sizes limit gender‐specific conclusions. Ziowbrowski and colleagues (2018) also found that adjusting for other comorbid psychiatric disorders attenuated ADHD risk in individuals with BED, but not in those with BN. ADHD prevalence estimates in these populations range from 15%–54% for BN and 10%–36% for BED (Appolinario et al. 2022; R. C. Kessler et al. 2013), notably higher than the 3%–7% in the general adult population (Song et al. 2021). This suggests an elevated ADHD prevalence, particularly in BN.

In clinical studies, ADHD rates appear more variable, potentially due to smaller sample sizes and because screening tests often yield higher ADHD prevalence than detailed assessments (Adamis et al. 2022). ADHD positive screening rates do not significantly vary across ED populations, nor across patients with BED and obesity compared to those with obesity alone (Ferre et al. 2017; Müller et al. 2012; Svedlund et al. 2017). This may be because rates of ADHD are also elevated in patients with obesity (Cortese et al. 2016). However, ADHD traits were significantly more elevated in BN and BED than in AN‐R (Sala et al. 2018; Svedlund et al. 2017).

There may be potentially confounding factors in these prevalence findings. Firstly, the impact of ADHD medication remains largely unexplored, as medication use was not reported or adjusted for in these studies. Secondly, Svedlund and colleagues (2021) observed reduced ADHD traits in BN patients after recovery, suggesting that some ADHD symptoms may stem from the ED rather than reflecting an underlying neurodevelopmental condition (Kinnaird and Tchanturia 2021). Retrospective childhood screening, as in Müller and colleagues (2012), could help circumvent this issue. However, the overall finding that ADHD is elevated in individuals with BED was supported by the opinions of researchers and healthcare professionals working within this field (Bray et al. 2022).

### 4.3 Clinical Differences of ADHD Patients With BN and BED

No studies fulfiling our eligibility criteria investigated differences between ADHD and non‐ADHD adults with BN or BED. However, Seitz and colleagues (2013) studied 15–35‐year‐olds with BN and found that those with childhood ADHD exhibited greater impulsivity, inattention, ED severity, and psychopathological symptoms than those without ADHD. While the sample included non‐adults, these findings likely extend to adult‐only samples, suggesting ADHD may be associated with clinically relevant differences, including increased ED severity, in BN.

### 4.4 Treatment Considerations for ADHD in BN and BED

Studies suggest stimulant medications may help reduce ADHD and ED symptoms. Case reports mostly used methylphenidate for patients with BN (Bhat et al. 2023; Drimmer 2003; Guerdjikova and McELROY 2013; Ioannidis, Serfontein, and Müller 2014; Jacquemet et al. 2021; Schweickert, Strober, and Moskowitz 1997; Sokol et al. 1999), which is the first‐line ADHD treatment for UK adults (NICE 2009) and has shown promise for BED (Quilty et al. 2019). One experimental study and three case reports administered lisdexamfetamine for ADHD patients with BN and BED (Bhat et al. 2023; Griffiths et al. 2024; Jeremias et al. 2024; Ruiz et al. 2019). Lisdexamfetamine, originally an ADHD medication, is US FDA‐approved for BED but remains an ADHD‐specific treatment in Europe (Himmerich, Bentley, and McElroy 2024). Two case reports also described prescribing dextroamphetamine (Dukarm 2005; Ioannidis, Serfontein, and Müller 2014), which lisdexamfetamine converts to.

Two additional studies described brain wave entrainment techniques for treating ADHD patients with BN: one used neurofeedback (Santarpia 2008) and the other employed sensory stimuli (Farber 2009). Both studies reported positive findings, although neither were blinded RCTs. No studies explored psychological therapy, despite its recommendation as a first‐line treatment for individuals with BED or BN (Giel et al. 2022), and an adjunct to ADHD medication (NICE 2009). Psychotherapy may be able to address underlying ADHD‐related behaviours contributing to the ED as well as teaching coping strategies. This makes it a promising options for managing comorbid ADHD and BN or BED, especially in patients unable to tolerate medications (Monteleone et al. 2022).

### 4.5 Diversity of Studies

Reviewed studies largely came from Western Europe and North America, particularly the USA. Few studies reported on participants' ethnicity or race. This presents generalisability challenges as autism, ADHD, and ED presentations vary across demographics (Culbert, Sisk, and Klump 2021; Faheem et al. 2022; Guagliardo et al. 2024; Napolitano et al. 2022). There is a pressing need for research that (explicitly) includes minoritised and underrepresented groups, including those from the global south, gender minorities, and non‐white populations (Halbeisen, Brandt, and Paslakis 2022).

Furthermore, this review reveals significant gaps in understanding sex differences due to limited and biased data. ADHD was elevated in BN and BED when controlling for gender in three cohort studies (Hudson et al. 2007; R. C. Kessler et al. 2013; Ziobrowski, Brewerton, and Duncan 2018). However, only one study examined sex differences (Brewerton and Duncan 2016), finding ADHD risk may be more pronounced in females with BN or BED, though male samples were too small for firm conclusions. Most other studies focused on females, with males underrepresented or absent. Diagnostic biases may have underrepresented autism and ADHD in women, further complicating research. For example, AdAS includes female‐specific autism features making it ideal for assessing autism in women, while AQ‐50 and RAADS‐R may lack sensitivity in this group (Dell'Osso et al. 2017). Similarly, ADHD measures such as CAARS better capture female presentations, while other measures are less nuanced (Young et al. 2020). Future studies should systematically address sex differences, ensure adequate male representation, and account for biases in diagnosing women.

### 4.6 Strengths and Limitations of This Review

This review provides an up‐to‐date, clinically focused summary of the current research on autism and ADHD in BN and BED. Unlike previous reviews, it incorporates qualitative perspectives, clinical differences, and treatment options, in addition to just prevalence. It avoids splitting ED patients by binge behaviours, acknowledging that BN and BED are often managed separately from AN, regardless of AN sub‐type. More broadly, it focuses on individuals with ED diagnoses over disordered eating populations, ensuring relevance to clinical practice. This review is designed to be accessible to clinicians, enabling practical applications in neurodivergent‐sensitive care, as well as providing a clear foundation to inform future research.

As this review focuses on adult patients, this limits generalisability to paediatric and adolescent populations and may exclude some informative studies. Additionally, it examined autism and ADHD prevalence and influence on EDs, rather than EDs prevalence or influence on autism or ADHD, making findings more applicable to ED‐focused clinicians and researchers, rather than those specialising in autism or ADHD. Studies on biological or genetic mechanisms were excluded due to limited immediate clinical relevance.

### 4.7 Future Research

Future research should examine the specific needs and clinical differences of Autistic and ADHD patients with BN and BED to improve treatment inclusivity. Developing targeted psychotherapeutic and educational interventions could address the distinct symptoms of these populations, potentially enhancing outcomes. Including more diverse populations in research will ensure findings are culturally relevant and more universally applicable.

### 4.8 Conclusion

Although autism and ADHD appear to be overrepresented in BN and BED, especially ADHD in BN, there is little research on how these conditions affect ED experiences or treatment outcomes. Treatment studies, largely case reports, primarily discuss ADHD medications, with limited exploration of non‐pharmacological options. To improve care, future studies should focus on understanding patient experiences and clinical differences, along with developing holistic, evidence‐based treatment strategies for Autistic and ADHD adults with BN and BED.

## Ethics Statement

The authors have nothing to report.

## Consent

The authors have nothing to report.

## Conflicts of Interest

The authors declare no conflicts of interest.

## Permission to Reproduce Material From Other Sources

The authors have nothing to report.

## Supporting information

Supporting Information S1

## Data Availability

Database search results and extraction forms will be made available on the OSF (https://osf.io/ydep7).
