# Supplementary material for: Autism, ADHD, and Their Traits in Adults With Bulimia Nervosa and Binge Eating Disorder: A Scoping Review
Source: Eur Eat Disord Rev. 2025 Jan 26;33(4):647–65. doi: 10.1002/erv.3177 (PMC12171673; doi:10.1002/erv.3177)
Supplement: Supplementary file 1 — Supporting Information S1 [file ERV-33-647-s001.docx]

**Autism, ADHD, and Their Traits in Adults with Bulimia Nervosa and Binge Eating Disorder: A Scoping Review**

Appendix 1. Eligibility criteria for papers included in this review.

| **Inclusion criteria** | **Exclusion criteria** |
| --- | --- |
| *Title and Abstract screening* | |
| - Involved human adults (18+ years) diagnosed with BN or BED. For qualitative studies, samples of carers, family members, health care professionals, or experts were also included. - Assessed for, or reported, diagnosed or suspected autism or ADHD, or autistic or ADHD traits (referred to simply as ‘autism’ and ‘ADHD’ for the rest of the criteria) in the sample. | - Studies on animals, children, other EDs, sub-clinical EDs or disordered eating behaviours were excluded. This was to ensure that literature selected is relevant to clinicians working with adult BN and BED patient populations. |
| *Full-text screening* | |
| - Reported relative prevalence of autism or ADHD in patients with BN or BED; experiences or perspectives of this group and other stakeholders; clinical differences of this patient group compared to patients without autism or ADHD; or intervention options for this group. - Prevalence papers had to report effect sizes (and confidence intervals) or p-values to be included. - Only studies with primary data or those that analyse secondary data (such as in cohort studies). - Peer-reviewed articles, pre-prints, and abstracts were all included. - The sample could be of any sex or ethnicity and samples with other comorbidities were not excluded. Studies could be from any location or year. | - Prevalence papers were excluded if they only reported raw percentages, without a comparison group and without reporting effect sizes or significance testing. - Studies were excluded if the target sample was aggregated with a wider sample, such as in a mixed ED sample or with children (17 and under). - Studies were also excluded if there was insufficient information in the abstract for extraction, and full text was not available in English, or an English version could not easily be generated. - No reviews, comment pieces, or theoretical papers. Reviews were set aside for hand-searching. - Studies which only investigated unidimensional autism or ADHD traits (e.g. impulsivity) were excluded. - Studies only looking at biological data were excluded, as these are unlikely to immediately inform clinical practice. |

*ADHD = attention-deficit/ hyperactivity disorder; BN = bulimia nervosa; BED = binge eating disorder; ED = eating disorder.*

Appendix 2. Full search strategy for each database.

| **Database** | **Search strategy** |
| --- | --- |
| Embase Classic + Embase | bulimia/ OR binge eating disorder/ OR obesity/ OR diabetic obesity/ OR morbid obesity/ OR sarcopenic obesity/ OR "bulimi*".kf,tw. OR “binge eating disorder*”.kf,tw. OR "obes*".kf,tw.  AND  autism/ OR asperger syndrome/ OR attention deficit hyperactivity disorder/ OR neurodiversity/ OR "autis*".kf,tw. OR "asperger*".kf,tw. OR attention deficit.kf,tw. OR adhd.kf,tw. OR “neurodiver*”.kf,tw.  NOT  ((exp animal/ or exp invertebrate/ or nonhuman/ or animal experiment/ or animal tissue/ or animal model/ or exp plant/ or exp fungus/) not (exp human/ or human tissue/)) |
| Ovid MEDLINE® ALL | bulimia nervosa/ OR binge-eating disorder/ OR obesity/ or obesity, morbid/ OR "bulimi*".kf,tw. OR “binge eating disorder*”.kf,tw. OR "obes*".kf,tw.  AND  exp Autism Spectrum Disorder/ OR Attention Deficit Disorder with Hyperactivity/ OR "autis*".kf,tw. OR "asperger*".kf,tw. OR attention deficit.kf,tw. OR ADHD.kf,tw. OR “neurodiver*”.kf,tw.  NOT  (exp animals/ not humans.sh.) |
| APA PsycINFO | bulimia/ OR binge eating disorder/ OR obesity/ OR "bulimi*".tw. OR “binge eating disorder*”.tw. OR "obes*".tw.  AND  exp autism spectrum disorders/ OR exp attention deficit disorder/ OR neurodiversity/ OR "autis*".tw. OR "asperger*".tw. OR attention deficit.tw. OR adhd.tw. OR “neurodiver*”.tw. |
| Clarivate Web of Science Core Collection | TS=(bulimi*) OR TS=(binge eating disorder*) OR TS=(obes*)  AND  TS=(autis*) OR TS=(asperger*) OR TS=(attention deficit) OR TS=(adhd) OR TS=(neurodiver*) |
| CENTRAL | MeSH descriptor: [Bulimia Nervosa] explode all trees OR MeSH descriptor: [Binge-Eating Disorder] explode all trees OR MeSH descriptor: [Obesity] explode all trees OR (bulimi*):ti,ab,kw OR (binge eating disorder*):ti,ab,kw OR (obes*):ti,ab,kw  AND  MeSH descriptor: [Autism Spectrum Disorder] explode all trees OR MeSH descriptor: [Attention Deficit Disorder with Hyperactivity] explode all trees OR (autis*):ti,ab,kw OR (asperger*):ti,ab,kw OR (attention deficit):ti,ab,kw OR (adhd):ti,ab,kw OR (neurodiver*):ti,ab,kw |
| Scopus (Elsevier) | ( TITLE-ABS-KEY ( bulimi* ) ) OR ( TITLE-ABS-KEY ( "binge eating disorder*" ) ) OR ( TITLE-ABS-KEY ( obes* ) )  AND  ( TITLE-ABS-KEY ( autis* ) ) OR ( TITLE-ABS-KEY ( asperger* ) ) OR ( TITLE-ABS-KEY ( "attention deficit*" ) ) OR ( TITLE-ABS-KEY ( adhd ) ) OR ( TITLE-ABS-KEY ( neurodiver* ) ) |

Appendix 3. PRISMA flow chart for this review.
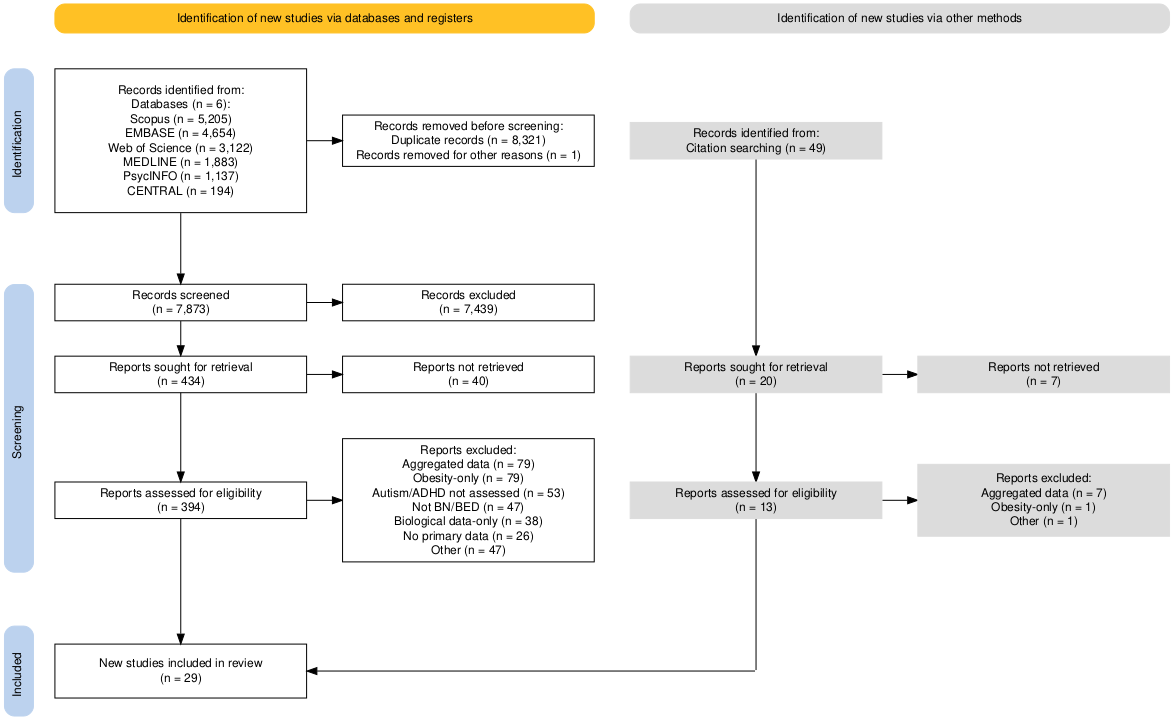
 *ADHD = attention-deficit/ hyperactivity disorder; BN = bulimia nervosa; BED = binge eating disorder.*

Appendix 4. Number of included publications from 1996 to 2024.

Appendix 5. Descriptions of qualitative studies.

|  | Study | Country | Sample | Age, years  *Mean±SD*  *(range)* | Gender *Women (%)* | Ethnicity *White (%)* | Findings  *Autism* | *ADHD* |
| --- | --- | --- | --- | --- | --- | --- | --- | --- |
| 14. | Bray et al. (2022) | USA | 14 experts (7 researchers, 6 clinicians and healthcare administrators, 1 unknown) | 55±10.2 (37-44) | 62 | 92 | *No expert’s thought autism was relevant to BED. Two experts mentioned autism in relation to ADHD, BED, and sensory input.* | *Thirteen experts considered ADHD to be relevant to BED. Five discussed the extent of this relevance. Six experts described frequent comorbidity between the ADHD and BED. Seven experts discussed the potential nature of this relationship, including relevant mechanisms. Seven experts discussed the use of stimulant medication with mixed views. Four participants discussed issues around detecting and treating this comorbidity. Three participants reported seeing a lot of untreated ADHD in patients with BED. Three participants reported benefits to patients if they can get diagnosed with ADHD when applicable.* |

*ADHD = attention-deficit/ hyperactivity disorder; BED = binge eating disorder; SD = standard deviation.*

Appendix 6: Summary of intervention and case report findings.

|  | BN | BED |
| --- | --- | --- |
| Autism | No studies. | An autistic patient with BED was successfully treated for depression with sertraline and aripiprazole. |
| ADHD | Eighteen female patients with ADHD and BN were treated with stimulant medications, mostly methylphenidate or Adderall to successfully reduce or extinguish ADHD or BN symptoms. One patient was also treated with audio-visual entrainment to successfully reduce their Ritalin dose. Ten patients were treated with neurofeedback, resulting in self-report positive changes, statistically significant reductions in two out of six ED symptoms, but no changes in brain waves. | Two patients with ADHD and BED were treated with methylphenidate, leading to weight loss, decreased severity of pathology, and reduction in binge episodes. An open label lisdexamfetamine trial of 41 adults with BED found that bingeing frequency reduced in three out of four patients with comorbid ADHD, but that overall ADHD symptoms did not decrease in a subset of 14 patients who positively screened for ADHD. |

*ADHD = attention-deficit/ hyperactivity disorder; BN = bulimia nervosa; BED = binge eating disorder; ED = eating disorders.*
